# Traditional Chinese medicine and plant-derived bioactive compounds as sustainable alternatives to antibiotics in bovine mastitis: a review

**DOI:** 10.3389/fvets.2025.1642647

**Published:** 2025-09-22

**Authors:** Xuewei Fan, Abdul Qadeer, Mohammed Asiri, Fuad M. Alzahrani, Khalid J. Alzahrani, Khalaf F. Alsharif, Muhammad Zahoor Khan, Xin Jiang

**Affiliations:** ^1^Heilongjiang Agricultural Economy Vocational College, Mudanjiang, China; ^2^Department of Cell Biology, School of Life Science, Central South University, Changsha, China; ^3^Department of Clinical Laboratory Sciences, College of Applied Medical Sciences, King Khalid University, Abha, Saudi Arabia; ^4^Department of Clinical Laboratories Sciences, College of Applied Medical Sciences, Taif University, Taif, Saudi Arabia; ^5^College of Agriculture and Biology, Liaocheng University, Liaocheng, China

**Keywords:** mastitis, antimicrobial resistance, antibiotics, inflammatory changes, bioactive compounds, traditional Chinese medicine

## Abstract

Bovine mastitis, an inflammatory condition of the mammary glands caused by diverse etiological agents, represents a significant economic challenge to the global dairy industry, resulting in annual losses of approximately $35 billion. While antibiotic therapy remains the conventional intervention for both prophylaxis and treatment, the increasing prevalence of antimicrobial resistance (AMR), particularly the emergence of multidrug-resistant and methicillin-resistant strains, has compromised therapeutic efficacy. These developments pose substantial concerns regarding milk safety and public health implications. Consequently, research attention has shifted toward alternative therapeutic modalities, encompassing phytotherapeutic interventions, nutritional modifications, and traditional Chinese medicine (TCM). Numerous plant species demonstrate significant antimicrobial properties while maintaining favorable safety profiles for humans, animals, and ecological systems. Complementary therapeutic approaches, including acupuncture and traditional herbal formulations, have exhibited promising potential in enhancing treatment outcomes and improving milk quality parameters. This review synthesizes current evidence on the integration of traditional Chinese medicine and plant-derived bioactive compounds into sustainable, holistic strategies for mastitis management, with implications for animal welfare, economic sustainability, and public health safety.

## 1 Introduction

The dairy industry is a fundamental pillar of global agricultural systems, contributing significantly to food security and economic stability worldwide (1). Mastitis, an inflammatory condition affecting the mammary glands, is one of the most economically devastating diseases in dairy production, characterized by distinct pathological alterations in mammary tissues accompanied by pronounced physical and chemical modifications in milk composition (2–4). This complex, multifactorial disease predominantly affects dairy cattle during the periparturient period, resulting from intricate interactions among host susceptibility factors, pathogenic microorganisms, and environmental management practices (5–7). The etiology of mastitis encompasses a diverse spectrum of more than 200 microbial agents, with Gram-positive and Gram-negative bacteria serving as the primary causative pathogens. At the same time, additional contributing factors include udder morphology, animal age, genetic predisposition (8–11), and environmental conditions (5, 12).

The economic ramifications of mastitis on the global dairy sector are profound, with conservative estimates indicating annual losses of approximately $35 billion worldwide (13). Regional economic assessments reveal similarly substantial impacts, with the United States sustaining approximately US$2 billion in annual losses, Canada experiencing Can$400 million (US$318 million) in economic damage, and China reporting financial losses ranging between 15 and 45 billion CNY (14, 15). These comprehensive financial impacts encompass multiple direct and indirect costs, including diminished milk yield, mandatory milk disposal due to antibiotic residues, veterinary intervention expenses, premature culling of chronically infected animals, and occasional mortality (5, 16–19). Detailed economic analyses reveal that approximately 60% of losses are attributable to decreased milk production, 16% to increased labor requirements, 9% to discarded milk, 7% to elevated animal replacement costs, 4% to reduced milk market value, 3% to medication expenses, and 1% to veterinary consultation fees (20).

The predominant bacterial pathogens associated with mastitis include *Staphylococcus aureus* (*S. aureus*), *Streptococcus agalactiae* (*S. agalactiae*), *Streptococcus uberis* (*S. uberis*), *Escherichia coli* (*E. coli*), and *Klebsiella pneumoniae* (*K. pneumoniae*) (21, 22). A growing concern is the rising incidence of antimicrobial resistance (AMR) among these pathogens, as documented in various global studies. Research from Ethiopia and Estonia has revealed high rates of penicillin-resistant *S. aureus* and coagulase-negative *staphylococci* (23, 24). At the same time, investigations in West Bengal, India, have identified Gram-negative bacteria resistant to *β*-lactams and tetracyclines (25). Comparable resistance patterns have been systematically documented in Central Mexico, where coagulase-negative *Staphylococci* represented 42% of udder pathogens, followed by *Streptococci* at 17%. Notable isolates included *S. aureus, Brevibacterium stationis* (*B. stationis*), *Brevibacterium conglomeratum* (*B. conglomeratum*), and *Raoultella* species, each comprising 8% of the total isolates. Critically, 72.7% of these isolates demonstrated multidrug resistance to three or more antimicrobial agents, with the highest resistance frequencies observed against penicillin, clindamycin, and cefotaxime (26). Parallel studies in Southern Taiwan revealed that *E. coli* isolates exhibited complete resistance to cloxacillin (100%) and demonstrated moderate resistance (50%) to tetracycline, neomycin, gentamicin, ampicillin, ceftriaxone, cefotaxime, and ceftazidime. Approximately 70% of isolates displayed resistance to at least two distinct antibiotics. In comparison, 28.1% harbored both AmpA and AmpC resistance genes simultaneously, with blaTEM representing the most frequently detected beta-lactamase gene, followed by blaCMY, blaCTX, blaSHV, and blaDHA (27). Advanced whole-genome sequencing analyses conducted in Canada on *S. uberis* and *S. dysgalactiae* isolates have revealed direct correlations between specific AMR genes and elevated minimum inhibitory concentrations (MICs), particularly for tetracyclines and lincosamides. In contrast, subclinical isolates continued to harbor AMR genes acquired through horizontal gene transfer mechanisms, emphasizing their critical role in resistance dissemination within dairy herds (28).

The evolutionary development of AMR, initially documented with penicillin-resistant *Streptococcus pneumoniae* (*S. pneumoniae*), occurs through sophisticated mechanisms involving the horizontal transfer of resistance genes via mobile genetic elements, including bacteriophages, plasmids, naked DNA, and transposable elements (29). While antimicrobial intervention remains indispensable for maintaining economic viability, ensuring animal welfare, and preserving mammary gland health in commercial dairy operations, the emergence and proliferation of resistant bacterial strains constitute a significant threat to global public health, food security, and sustainable agricultural development (29, 30). This concerning development has prompted increased interest in alternative therapeutic approaches (15), including nutritional interventions, bioactive compound therapies, and evidence-based plant-derived treatments (31–35).

From a broader public health perspective, the increasing incidence of bovine mastitis frequently necessitates intensive antibiotic usage, consequently elevating the risk of antibiotic residues in milk products and contributing to the global AMR burden, ultimately increasing healthcare costs and threatening therapeutic efficacy. To address these multifaceted challenges and reduce dependence on conventional antimicrobials, researchers worldwide are systematically investigating alternative treatment strategies, including homeopathic approaches, with a rigorous emphasis on ensuring therapeutic efficacy and safety for both animals and consumers (36).

Medicinal plants represent a vast repository of bioactive compounds with demonstrated therapeutic potential, containing diverse phytochemical constituents that exhibit beneficial effects on human and animal health. The encouraging empirical evidence supporting plant-based therapies has generated substantial scientific interest in exploring these natural substances for developing innovative therapeutic interventions. Plant extracts and essential oils, renowned for their broad-spectrum antimicrobial properties, represent up-and-coming alternatives that are generally recognized as safe for animals, humans, and environmental systems (30). Plants synthesize a diverse array of secondary metabolites as integral components of their natural defense mechanisms, many of which possess potent antimicrobial properties and have maintained significant roles in traditional medicinal systems throughout human history. The antimicrobial efficacy of plant-derived compounds is primarily attributed to diverse classes of bioactive phytochemicals, including flavonoids (such as quercetin, kaempferol, and catechins), alkaloids (including berberine, quinine, and morphine), terpenoids and terpenes (encompassing monoterpenes, sesquiterpenes, and triterpenes), phenolic acids (such as gallic acid, caffeic acid, and ferulic acid), saponins, tannins, and essential oil components (including thymol, carvacrol, eugenol, and linalool). These phytochemicals exhibit antimicrobial activity through multiple mechanisms, including disruption of bacterial cell wall synthesis, interference with cytoplasmic membrane integrity, inhibition of nucleic acid synthesis, disruption of metabolic pathways, and interference with bacterial communication systems (quorum sensing). Flavonoids demonstrate antimicrobial efficacy by forming complexes with extracellular proteins and bacterial cell walls, while alkaloids exert their effects through DNA intercalation and enzyme inhibition. Terpenoids compromise membrane integrity and interfere with respiratory processes, whereas phenolic compounds disrupt cellular metabolism and protein function (37). The therapeutic application of traditional Chinese medicine (TCM) in mastitis management, including established formulations such as Yanghe decoction (38), Danggui buxue decoction (39), Medulla tetrapanacis water extract (31), and Red ointment (40), has demonstrated considerable clinical success and therapeutic efficacy (41–44). Beyond TCM, bioactive phytocompounds have emerged as promising alternatives to conventional antibiotics for treating bovine mastitis (15, 45–50). This review critically evaluates the current evidence base for the therapeutic application of TCM and plant-derived bioactive compounds in bovine mastitis management.

## 2 Methodology for literature search

This review aims to explore the role of TCM and plant-derived bioactive compounds in the treatment of mastitis. To achieve this, a comprehensive literature search spanning 2014–2025 (11 years) was conducted using reputable databases including Google Scholar, PubMed, Web of Science, X-MOL, and additional Chinese databases (CNKI, Wanfang, VIP). Keywords such as TCM, plant-derived bioactive compounds, Chinese herbal medicine, bovine mastitis, udder health, herbal formulations, mastitis risk factors, and antimicrobial resistance were employed to find relevant studies. The inclusion criteria for this review were as follows: articles published between 2014 and 2025 were considered, with a specific focus on the application of TCM and plant-derived bioactive compounds for mastitis treatment. Studies published as book chapters, conference papers, abstracts, or in newspapers were excluded from this review.

## 3 Mastitis classification and possible risk factors

**Figure 1:**
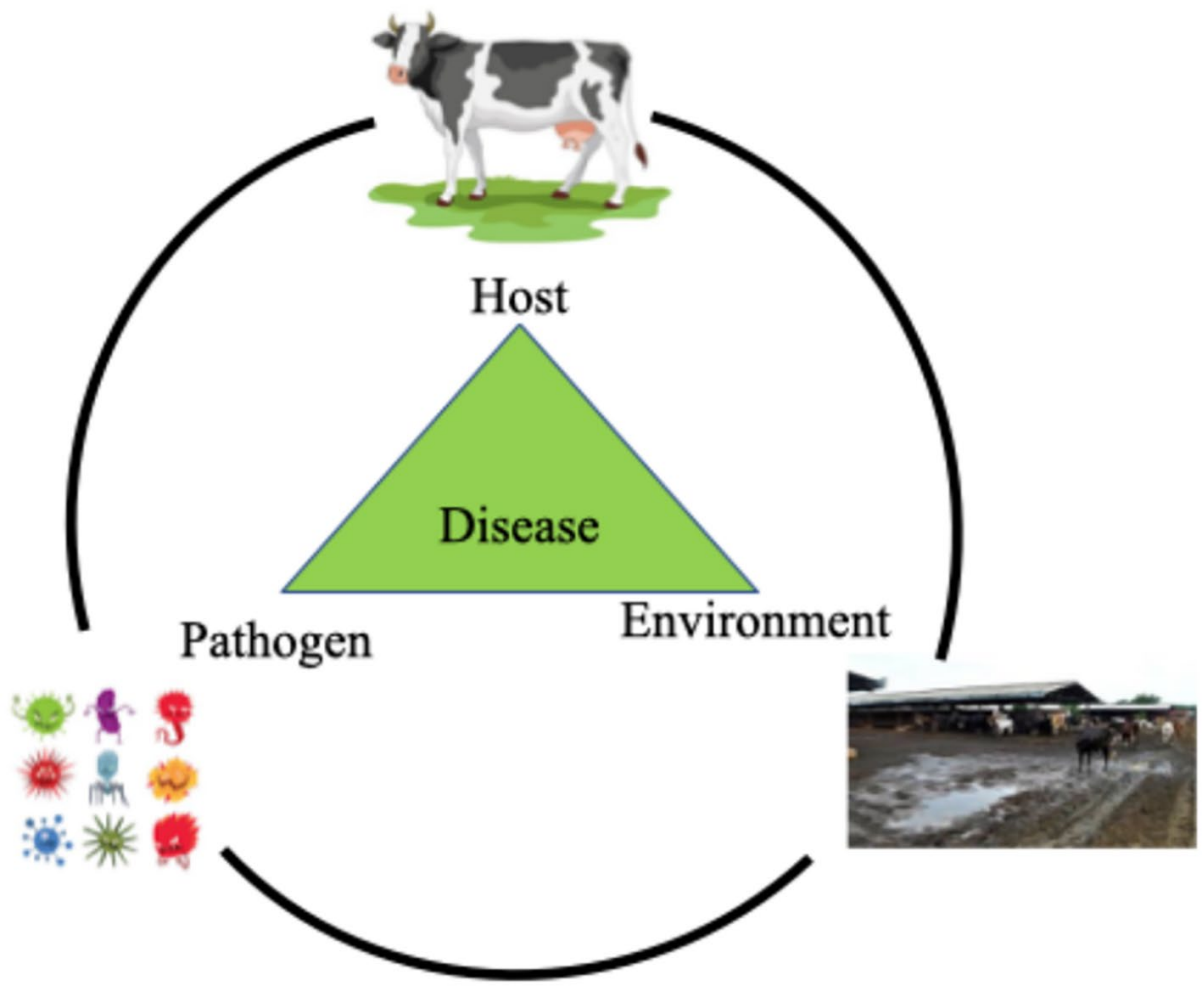
Host-pathogen-environment interactions and their association with mastitis. This diagram illustrates the disease triangle model, showing how mastitis develops through the interaction of three key factors. The host (in this case, a cow) represents the susceptible animal, with factors such as age, immunity, and genetics influencing susceptibility to mastitis. The pathogen shows various disease-causing agents, including bacteria, viruses, fungi, and parasites, that can cause mastitis. The environment (farm setting) depicts conditions influencing transmission, such as temperature, humidity, housing, milking practices, and sanitation. The central green triangle represents mastitis occurring at the intersection where all three factors align - when a susceptible host encounters a virulent pathogen under favorable environmental conditions (85).

The etiological agents of mastitis are delineated into three distinct categories based on the nature and origin of the causative pathogens: contagious, environmental, and opportunistic agents (Figure 1) (51).

### 3.1 Type of mastitis

**Figure 2:**
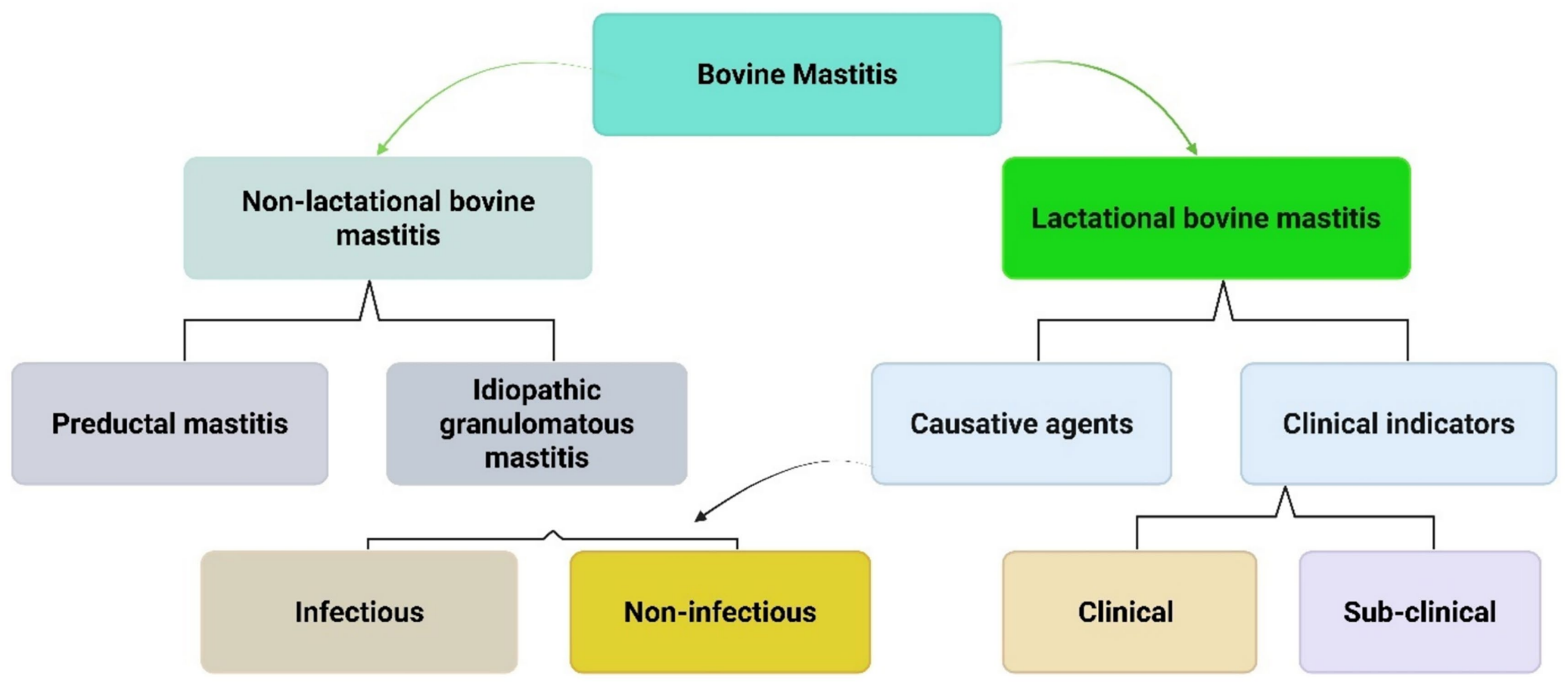
Classification of mastitis types. This flowchart classifies bovine mastitis (mammary gland inflammation in cattle) into two main categories. Non-lactational mastitis occurs when cows aren’t producing milk and includes preductal and idiopathic granulomatous types. Lactational mastitis occurs during milk production and is classified by cause (infectious vs. non-infectious) and symptoms (clinical with visible signs vs. sub-clinical with hidden infection).

Broadly, mastitis can be categorized into two types: lactational and non-lactational mastitis (as shown in Figure 2). The most common form is lactational mastitis, which typically occurs during breastfeeding. This condition is infectious and presents with localized pain and swelling, accompanied by systemic symptoms. Although it can develop at any time during the lactation period, it most frequently occurs during the second or third week of postpartum. Non-lactational mastitis includes two primary forms: idiopathic granulomatous and periductal mastitis. Periductal mastitis, though rare, can affect non-lactating individuals, particularly those of reproductive age. It’s often linked to bacterial infection (52).

Lactational mastitis can be further classified as either clinical or subclinical intramammary inflammation, based on the presence or absence of visible symptoms. The primary causative agents in clinical cases are Gram-negative bacteria, with *E. coli* being the most common (53). Clinical condition is primarily categorized into two main presentations: acute and chronic. Acute mastitis is characterized by overt inflammatory signs, including erythema, localized hyperthermia, and tissue tumefaction at the affected site. In severe manifestations, systemic complications may emerge, including pyrexia, septicemia, and abscess formation. Conversely, chronic mastitis exhibits a more insidious progression, typically characterized by recurrent infections and progressive tissue deterioration (2).

In contrast, subclinical mastitis (SCM) represents a pathogenic infection that proceeds without overt clinical manifestations or systemic symptomatology. However, it is distinguished by diminished milk production, compromised quality parameters, and a marked increase in somatic cell count (SCC) (54, 55). This form is predominantly associated with Gram-positive bacterial infections, most notably *S. aureus* (53). Clinical mastitis is generally easy to diagnose due to visible symptoms, but SCM lacks obvious signs of inflammation, making it more challenging to detect. Diagnostic tools such as the California mastitis test (CMT), elevated SCC in milk, and microbial isolation and culture from milk samples aid in identifying SCM. Early detection is crucial in the dairy industry to minimize financial losses. Numerous microorganisms, primarily bacteria, have been identified as the causative agents of mastitis (56). SCC is a key marker for evaluating udder health and serves as a reliable method for detecting mastitis by quantifying immune cells, such as neutrophils, lymphocytes, and macrophages, in milk (57). When SCC levels exceed 200,000 cells/ml, it often indicates a bacterial infection. Both clinical and SCM can lead to significant changes in SCC level, highlighting the ongoing inflammatory response in the udder (58).

### 3.2 Pathogenic factors

Environmental pathogens constitute a diverse group of microorganisms that originate from multiple reservoirs within the agricultural environment, including bedding substrates, arthropod vectors, housing infrastructure, and the bovine enteric microbiome, with *E. coli* representing a predominant example (59). The proliferation and transmission of these pathogens are significantly influenced by suboptimal husbandry conditions, including excessive stocking density, inadequate floor sanitation, insufficient ventilation systems, and elevated ambient temperatures coupled with high relative humidity (60). Contagious pathogens, primarily represented by *S. aureus* and *S. agalactiae*, exhibit host-adapted characteristics and are transmitted through direct inter-animal contact or via contaminated milking apparatus (2). Opportunistic pathogens demonstrate dual behavioral characteristics, functioning as either contagious or environmental agents depending on circumstances, and typically exploit periods of immunocompromised host status to establish intramammary infections (51).

Recent epidemiological studies have documented increased morbidity associated with mycotic mastitis in bovines. Notable fungal pathogens include zoonotic yeasts such as *C. albicans* and *Kodamaea ohmeri*, along with other Candida species: *C. guilliermondii*, *C. famata*, *C. tropicalis*, *C. colliculosa*, *C. krusei*, *C. rugosa*, *C. glabrata*, *C. parapsilosis*, and *C. inconspicua*. Additional fungal agents encompass Trichosporon species, *Rhodotorula glutinis*, *Saccharomyces fragilis*, *Pichia kudriavzevii*, and *Cyberlindnera rhodanensis*. Mold species, including *Aspergillus amstelodami*, *A. fumigatus*, and *Geotrichum candidum*, have also been implicated (61). Furthermore, yeast-like algae, specifically *Prototheca zopfii* and *Prototheca blaschkeae*, have been identified as causative agents (21). Yeast-like algae, including *Prototheca zopfii* and *Prototheca blaschkeae*, have also been implicated (62).

Viral infections contribute significantly to mastitis pathogenesis through direct and indirect mechanisms (63). Direct viral mastitis occurs with bovine herpesvirus 1 and 4 (clinical and subclinical presentations, respectively) (64–66), parainfluenza virus, and foot-and-mouth disease virus (67–69). Indirect viral contributions result from teat epithelial lesions caused by bovine herpesvirus 2, cowpox virus, pseudo-cowpox virus, vesicular stomatitis virus, papillomavirus, and bovine leukemia virus, which compromise barrier defenses and predispose to secondary bacterial invasion (21, 63, 70–72).

### 3.3 Non-pathogenic factors

Mechanical trauma associated with automated milking systems represents a critical predisposing factor, as it disrupts the anatomical integrity of the udder quarter. Specifically, compromised keratin plug formation and mucosal damage to the teat sinus create portals of entry for pathogenic microorganisms (21, 73). Suboptimal milking hygiene protocols demonstrate a significant positive correlation with mastitis incidence, emphasizing the importance of standardized sanitation procedures (21). In addition to mechanical factors, genetic and phenotypic characteristics substantially influence mastitis susceptibility through multiple interconnected pathways. Breed-specific variations reveal differential susceptibility patterns, with high-producing Holstein-Friesian cattle exhibiting increased vulnerability relative to medium-yielding Jersey cattle (74), while low-yielding Rendena cattle demonstrate superior disease resistance (60). Moreover, parity effects indicate a heightened susceptibility in multiparous compared to primiparous animals, reflecting cumulative exposure and potential immunological changes (75).

Beyond genetic predisposition, immunological determinants play a fundamental role in disease susceptibility through variations in cytokine expression profiles and humoral immune responses (76). Critical immune effector mechanisms include antimicrobial peptides (such as lysozyme and lactoferrin), cellular immune components (macrophages and neutrophils), and hormonal receptor expression patterns, which collectively modulate the host defense capacity (77). Concurrently, anatomical predispositions include specific udder conformations, particularly pendulous udder structure and funnel-shaped teat morphology, which facilitate pathogen entry and retention (12). Additionally, age-related physiological changes in geriatric animals contribute to increased susceptibility through progressive teat canal dilation and enhanced mammary epithelial permeability (60).

Temporal physiological changes further complicate these intrinsic factors, as the periparturient transition period represents a critical vulnerability window characterized by profound metabolic and immunological alterations. During this phase, nutritional status has a significant influence on mastitis susceptibility, particularly given the substantial metabolic demands associated with colostrum synthesis and lactogenesis in dairy cattle (12). Consequently, a negative energy balance can precipitate deficiencies in essential proteins, trace minerals, and vitamins that are fundamental to optimal immune function (78). Therefore, maintaining an adequate nutritional status, including sufficient selenium, iron, copper, zinc, cobalt, chromium, essential amino acids, and vitamins A, E, and C, is paramount for both mastitis prevention and sustained lactational performance (79).

## 4 Factors involved in antibiotic resistance

The primary approach to treating bovine mastitis involves the use of antibiotics. However, the effectiveness of this treatment is diminishing due to the rising incidence of antibiotic-resistant bacteria, which is now recognized as a significant global health concern (80). While antimicrobials have considerably improved animal health and yield, the improper or unnecessary use of antimicrobials in food-producing animals is believed to play a significant role in the development of AMR (81). Moreover, the presence of residues in milk poses potential risks to both animal and human health (82). The utilization of antimicrobial agents in animal husbandry has been a longstanding practice, primarily for therapeutic purposes and occasionally for production enhancement. These agents are also employed prophylactically to prevent infections. In dairy cattle management, antimicrobials are predominantly used to control mastitis, a prevalent and economically significant disease, during two crucial phases: lactation therapy and dry cow therapy (30).

Specifically, lactation therapy presents a significant challenge as antimicrobial use necessitates extended milk withdrawal periods due to the risk of drug residues. These residues present multiple concerns for human health, including potential adverse reactions in hypersensitive individuals, promotion of antimicrobial resistance, and interference with dairy product manufacturing processes (30). In contrast, dry cow therapy, which involves administering long-acting antimicrobials to all mammary quarters at the end of lactation, serves both therapeutic and preventive purposes. Although this approach has been fundamental to mastitis control programs, concerns about increasing AMR have prompted many nations to re-evaluate the use of prophylactic antimicrobials in livestock (83).

Unfortunately, the excessive and inappropriate use of antibiotics in mastitis treatment has substantially contributed to the emergence of antimicrobial and multidrug resistance, thereby complicating disease management (84). Consequently, prolonged or excessive antibiotic administration disrupts the internal microbial equilibrium, promotes the development of resistance, and results in antibiotic residues in milk (85). The underlying bacterial resistance mechanisms encompass the presence of resistant variants, selective reproductive advantages under antibiotic pressure, and the heritability of resistance traits, potentially leading to resistant strain dominance within populations (86).

A prominent example of this resistance challenge is *S. aureus*, a major pathogen of mastitis, which exemplifies this problem through its persistent and recurrent infections that often resist treatment. Notably, methicillin-resistant *Staphylococcus aureus* (MRSA) was first identified as a causative agent of mastitis in cows in 1972 (87). These methicillin-resistant *S. aureus* strains, which carry the mecA gene encoding penicillin-binding protein 2a, demonstrate resistance to all *β*-lactam antibiotics, including penicillin, cephalosporins, and carbapenems. Furthermore, MRSA frequently exhibits resistance to multiple antibiotic classes, including aminoglycosides, macrolides, tetracyclines, and fluoroquinolones (30).

The rapid evolution of bacterial resistance, driven by the widespread use of antimicrobials, has emerged as a global public health crisis (88). This situation is further exacerbated by limited research and development of new antimicrobial agents. Addressing this challenge requires a comprehensive understanding of resistance mechanisms and the development of novel antimicrobial strategies (30). As a result, there is an urgent need to identify and develop alternative therapeutic approaches that address these concerns while maintaining high standards of animal welfare and public health (89). In response to this critical need, the development of new alternative therapies and treatments presents a significant opportunity that requires collaborative efforts between veterinary practitioners and researchers. Traditional Chinese herbs offer several advantages over conventional antibiotics, including reduced side effects, a lower risk of bacterial resistance, minimal toxicity, and negligible residue levels. Additionally, they are used in the treatment of mastitis, as seen in the use of TCM and its extracts in treating mastitis (89–91).

## 5 Use of TCM and plant-derived bioactive compounds as an alternative treatment for bovine mastitis

### 5.1 TCM formulations and therapeutic approaches

TCM has garnered significant attention as an effective alternative to conventional antibiotic treatments for mastitis, demonstrating therapeutic efficacy while minimizing risks associated with antimicrobial resistance and secondary complications. The comprehensive antibacterial, anti-inflammatory, immunomodulatory, and antioxidant properties of TCM, developed over centuries of use, position it as a viable alternative therapy for mastitis treatment (32, 41, 92, 93). Building upon this foundation, numerous TCM formulations have been systematically introduced for the treatment of various types of mastitis (94). These include a comprehensive range of therapeutic options, such as Chai Hu Qing Gan Tang (95), Yanghe decoction (96), and Chaihu Qinggan (38). Furthermore, other notable formulations include Tuoli Tounong Decoction (97), Yiqi Heying (98) and Gong Ying San (99).

Among the most extensively studied traditional formulations, Jingfang Granules (JF’s) demonstrate remarkable efficacy in treating LPS-induced mastitis through multiple therapeutic pathways. Specifically, these granules operate through nuclear factor κB (NF-κB), phosphatidylinositol 3-kinase (PI3K), Akt, mitogen-activated protein kinase/Extracellular signal-regulated kinase (MAPK/ERK), p38, and nucleotide-binding oligomerization domain, leucine-rich-containing family, pyrin domain-containing-3 (NLRP3) signaling cascades. Moreover, they maintain milk barrier integrity through the regulation of tight junction proteins and prevent cell apoptosis by modulating Bcl-2 and Bax expression (100). In parallel, Qicao Rukang powder has demonstrated comparable effectiveness in treating SCM, showing notable improvements in SCC, milk composition, and bacteriological cure rates. The powder’s therapeutic efficacy is attributed to its diverse active constituents, including polysaccharides, saponins, flavonoids, and terpenoids (101). Complementing these oral formulations, Pulsatilla saponin B4 injection protocols have shown significant effectiveness in treating clinical mastitis. These protocols achieve therapeutic benefits by reducing SCC, eliminating pathogenic bacteria, and lowering inflammatory markers, including CRP, SAA, HP, and various pro-inflammatory cytokines (102). Notably, the integration of traditional approaches has shown auspicious results when combined with modern therapeutic techniques. For instance, combined therapy using intramammary antibiotics and complementary acupuncture has demonstrated substantial efficacy in reducing bovine mammary inflammation in cases of SCM. This innovative approach, which targets specific points on affected mammary quarters, resulted in a significant reduction of N-acetyl-beta-D-glucosaminidase (NAGase) activity, thereby indicating improved healing of mammary epithelial cells (103).

Expanding beyond traditional Chinese formulations, a comprehensive evaluation of Tibetan herbal medicines has revealed additional therapeutic options. These include *Swertia bimaculata*, *Gentiana urnula*, *Uncaria rhynchophylla*, *Aconitum flavum*, *Dracocephalum tanguticum*, and *Lagotis brachystachy*, all of which demonstrated significant antibacterial activity against mastitis-causing *Staphylococcus* strains. Particularly noteworthy is *Lagotis brachystachy*, which demonstrated exceptional efficacy against MDR strains (104). Ultimately, clinical studies have provided robust validation of the benefits of TCM in the treatment of acute mastitis. These investigations have demonstrated significant improvements across multiple parameters, including clinical effectiveness, lactation rates, symptom relief, quality of life, and emotional well-being. Collectively, these findings provide strong evidence supporting the efficacy of TCM external therapy in both symptom alleviation and promoting recovery (105).

### 5.2 Plant-derived bioactive compounds

Contemporary research has increasingly focused on identifying and characterizing plant-derived bioactive compounds with substantial therapeutic potential for managing mastitis. Among the most promising candidates are *Dimethyl itaconate*, Polydatin, Sinomenine hydrochloride, and Jiawei Tounong powder, which have demonstrated significant efficacy through various molecular mechanisms (106–109). Of particular significance is Shikonin (SHI), a bioactive natural naphthoquinone constituent extracted from *Lithospermum erythrorhizon*, which shows remarkable anti-inflammatory and antimicrobial properties. Initially utilized in TCM for treating wounds and various skin conditions, SHI has subsequently emerged as a viable therapeutic alternative to conventional antibiotics in managing inflammatory conditions, most notably lipopolysaccharide-induced mastitis. The underlying mechanism of action involves the systematic inhibition of the NF-κB signaling pathway through the targeted suppression of p-IκB*α* and p-p65 proteins, thereby achieving a substantial reduction in pro-inflammatory cytokines, including TNF-α, IL-1β, and IL-6 (110). Furthermore, SHI effectively alleviates oxidative stress through the activation of the Nrf_2_/HO1 signaling pathway (111).

Complementing these findings, comprehensive essential oil studies have revealed significant bacteriostatic activity of traditional extracts, particularly those from lemon balm and peppermint oil, against prevalent mastitis pathogens, including *S. aureus* and *E. coli* (112). In parallel, Sodium houttuynia (SH), derived from *Houttuynia cordata*, has demonstrated considerable efficacy in inhibiting LPS-induced inflammatory responses in bovine mammary epithelial cells (bMECs). The therapeutic mechanism involves the sophisticated modulation of the NF-κB signaling pathway, resulting in markedly reduced pro-inflammatory cytokine expression (IL-1β, IL-6, TNF-*α*) and decreased levels of Toll-like receptor 4 (TLR4), inhibitor of nuclear factor kappa B (IκBα), and NF-κB p65 (113).

Building upon these observations, Zhang et al. (114) conducted comprehensive investigations into the protective effects of *Salvia miltiorrhiza* polysaccharides (SMPs) in *S. aureus*-induced mastitis models. Their findings convincingly demonstrated that SMP treatment significantly reduced bacterial load, inflammatory cell infiltration, and cytokine levels while simultaneously inhibiting activation of the NF-κB and MAPK pathways. These therapeutic effects were substantiated by notable histopathological improvements and significant reductions in MPO and NAGase activity (114). Correspondingly, quercetin, extracted from *Ligustrum lucidum*, has exhibited considerable promise in both the prevention and treatment of mastitis. Through sophisticated network pharmacological analysis, researchers identified seven active ingredients and 42 key molecular targets, with tumor necrosis factor (TNF), alpha serine/threonine kinase 1 (AKT1), and interleukin-6 (IL-6) serving as core therapeutic targets. Subsequent *in vivo* studies validated quercetin’s capacity to alleviate pathological changes and downregulate inflammatory markers through the modulation of the PI3K-AKT and NF-κB signaling pathways (115).

Furthermore, geraniol has emerged as an up-and-coming therapeutic alternative, demonstrating effective pathogen inhibition, probiotic enhancement, and maintenance of gut microbial diversity. Notably, geraniol treatment exhibited no detectable milk residues after four days of administration and, significantly, did not induce drug resistance during prolonged exposure periods (116). Concomitantly, *Taraxacum mongolicum* has been shown to exhibit substantial protective effects against *S. aureus*-induced mastitis through well-characterized anti-inflammatory mechanisms, including the targeted downregulation of TLR2 and the systemic inhibition of NF-κB and MAPK signaling pathways (117).

Of exceptional interest, Forsythiaside A (FTA) has established a pivotal role in mastitis treatment through multiple comprehensive studies (118–121). Recent research has successfully elucidated FTA’s sophisticated protective mechanisms, particularly its capacity to modulate mitophagy through the PINK1/Parkin signaling pathway. This pathway represents a crucial component for maintaining mitochondrial integrity, cellular energy production, and cell viability under conditions of mastitis-induced stress. FTA’s selective activation of mitophagy facilitates the targeted removal of dysfunctional mitochondria, thereby preserving mitochondrial integrity and reducing inflammatory responses. Additionally, FTA demonstrates remarkable effectiveness in lowering both cellular and mitochondrial reactive oxygen species (ROS), thereby mitigating oxidative damage, associated inflammation, and tissue injury. These synergistic mechanisms collectively contribute to reduced mastitis severity and improved dairy cow health and productivity (122).

Beyond these extensively characterized compounds, various other traditional therapeutic agents have demonstrated promising potential. Specifically, Tanshinone I and Tanshinone IIA/B exhibit significant inhibition of NF-κB activation in nMECs, proving particularly effective when combined with conventional antibiotics such as cephalosporins (123). Similarly, *Artemisia argyi* Leaves (ALE) have shown substantial therapeutic potential in LPS-induced mouse mastitis models, demonstrating the capacity to alleviate tissue damage, reduce oxidative stress, and regulate inflammation-associated gene expression (124). Moreover, Broadleaf Mahonia has been shown to exhibit significant anti-inflammatory properties by reducing pro-inflammatory cytokines, including IL-1β, CCL-5, and IL-6, in RAW264.7 cell cultures. This therapeutic effect is primarily mediated through systematic inhibition of NF-κB and MAPK signaling pathways, which represent crucial regulators of inflammatory responses. In cases of granulomatous lobular mastitis, preventive treatment with *Broadleaf mahonia* effectively reduces inflammation and promotes tissue homeostasis (125).

**Figure 3:**
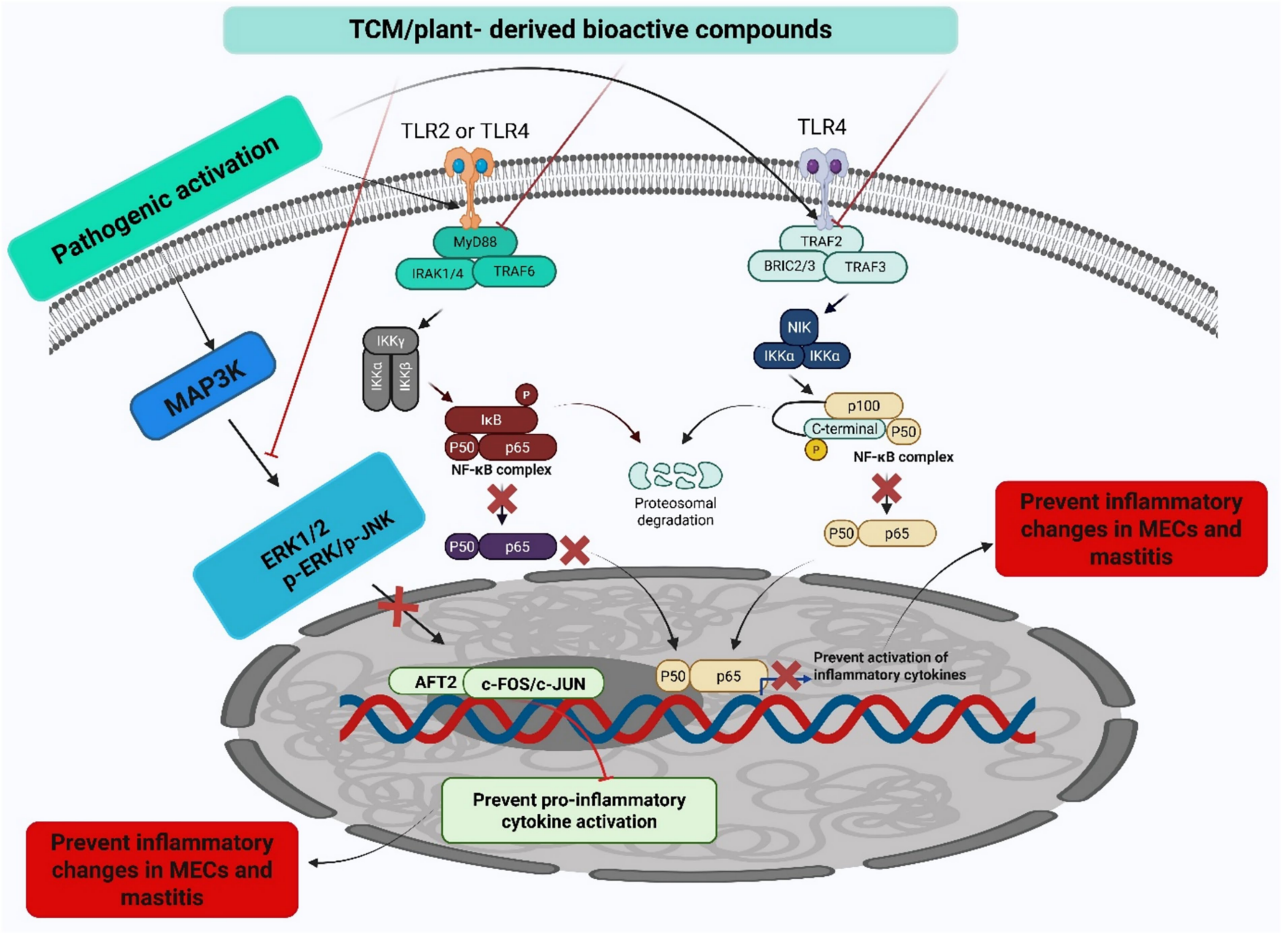
Anti-inflammatory mechanisms of TCM and plant-derived bioactive compounds. This figure illustrates the mechanism by which conventional TCM and plant-derived bioactive compounds exert anti-inflammatory effects by inhibiting key inflammatory signaling pathways, specifically the MAPK and NF-κB pathways, ultimately leading to the prevention of mastitis. The conceptual framework presented in this figure is adapted from findings reported in previously published literature (12, 77, 88).

**Figure 4:**
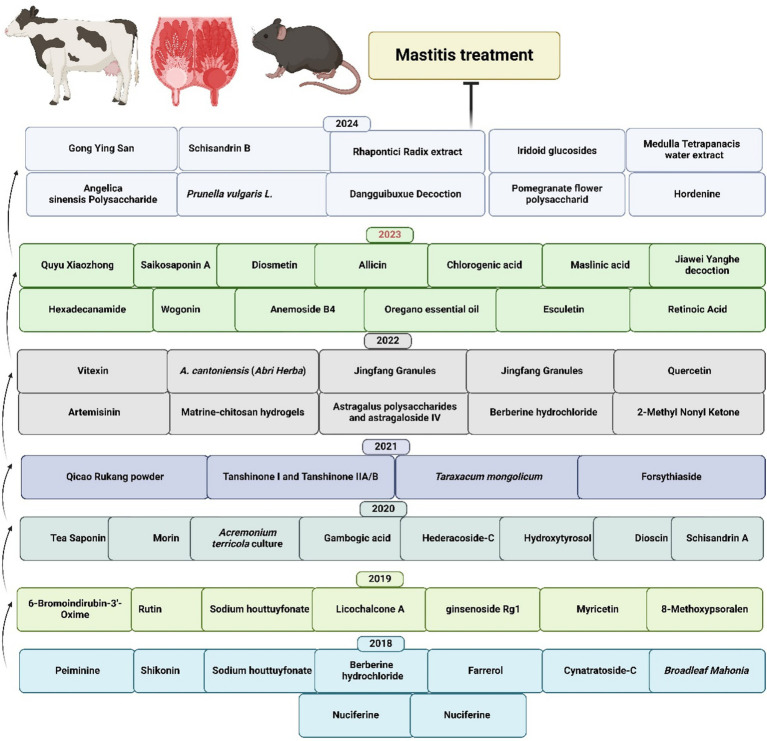
Recent advances in TCM and plant-derived bioactive compounds for mastitis treatment (31, 89, 99–101, 110, 113, 115, 117, 120, 123, 125, 130–178).

To comprehensively understand the underlying pathophysiological mechanisms, investigations into mastitis caused by *S. aureus* have revealed significant inflammatory responses characterized by elevated levels of IL-1β, TNF-*α*, and MPO activity, alongside increased ferroptosis markers such as Fe^2+^ and MDA levels. Decreased protective factors, including GSH, GPX4, and ferritin in mammary tissues, accompany these pathological changes (126). Remarkably, the strategic integration of traditional therapeutic approaches has yielded promising clinical results. In this context, Schisandrin B (SB) treatment has demonstrated considerable effectiveness in mitigating pathological changes by reducing both inflammation and ferroptosis. The underlying therapeutic mechanism involves the upregulation of SIRT1 and SLC7A11 expression, the inhibition of p53 and NF-κB activation, and the restoration of antioxidant defense systems. These therapeutic effects were confirmed through comprehensive histological analysis, demonstrating reduced tissue damage in SB-treated groups, thereby suggesting that SB’s therapeutic action occurs through the SIRT1/p53/SLC7A11 and NF-κB pathways (127). The molecular mechanisms underlying the protective effects of traditional Chinese medicine and plant-derived bioactive compounds against mastitis are schematically represented in Figure 3. Additionally, a comprehensive overview of research developments on traditional Chinese medicine and plant-derived bioactive compounds in mastitis therapy is summarized in Table 1 and Figure 4.

**Table 1:** Summary of research on TCM and plant- derived bioactive compounds for mastitis treatment (2014–2025).

TCM/plant- derived bioactive compounds	Biological effects	Research model	References
*Medulla Tetrapanacis* water extract	Decreased inflammatory cytokines (TNF-*α*, IL-6, and interleukin-1 beta), Protected blood-milk barrier integrity by enhancing protecting blood-milk barrier integrity and inactivate MAPK-signaling pathways to prevent inflammatory changes	HuMEC and rats	(31)
Danggui buxue decoction	The DD showed anti-inflammatory (suppressed TNF-*α*, IL-1β, IL-6, IL-8, TLR4 and NF-κB) and antibacterial activities Relieved oxidative stress by enhancing antioxidant response (inhibiting levels of malondialdehyde (MDA), nitric oxide (NO) content assay, and reactive oxygen species (ROS) followed by enhanced superoxide dismutase (SOD), total antioxidant capacity (T-AOC), and glutathione (GSH) activities) in LPS challenged BMECs.	BMECs	(89)
Shikonin (naphthoquinone constituent extracted from Chinese herb *Lithospermum erythrorhizon*)	Shikonin prevents LPS-induced mastitis by suppressing TNF-α, IL-1β, and IL-6 followed by inhibition of p-IκBα and p-p65, which are the critical proteins functioning in NF-kB signaling pathway	Mice	(110)
*Salvia miltiorrhiza* polysaccharides	*Salvia miltiorrhiza* polysaccharides prevent mastitis induced by *S. aureus* in rats. Significantly reduced bacterial load, inflammatory cell recruitment, and the expression of inflammatory cytokines like IL-1β, IL-6, and TNF-α. Inhibited the activation of NF-κB and MAPK signaling pathways.	Rats	(114)
Jingfang Granules (JF)	alleviated LPS-induced mastitis by mitigating inflammation, preserving the integrity of the blood-milk barrier, and modulating cellular apoptosis. JF decreased myeloperoxidase (MPO) activity and the expression of pro-inflammatory cytokines, including IL-1β, IL-6, and TNF-α. Reduced the protein levels of key inflammatory signaling molecules such as TLR4, P-NF-κB, NLRP3, ASC, Caspase-1, IL-1β, P-P38, P-ERK1/2, and P-AKT, indicating its regulatory effect on NF-κB, NLRP3, PI3K/AKT, and MAPK pathways. Enhanced the expression of tight junction proteins (ZO-1, Claudin-3, and Occludin), thereby improving the integrity of the blood-milk barrier.	Mice	(100)
*Artemisia argyi* leaves extract	Alleviated mammary gland lesions through its anti-inflammatory and antioxidant properties. Significantly reduced LPS-induced myeloperoxidase (MPO) activity and restored antioxidant enzymes like glutathione peroxidase (GSH-PX) and superoxide dismutase (SOD), while mitigating oxidative imbalance caused by nitric oxide overproduction. Down-regulated inflammatory factors IL6, TNFα, and IL1β, and modulated the TLR2/4 signaling pathway via MyD88. Inhibited the NF-κB signaling pathway by alleviating IκB degradation.	Mice	(124)
*Broadleaf Mahonia*	Broadleaf Mahonia treatment significantly reduced the expression of inflammatory markers, including IL-1β, CCL-5, and IL-6, in RAW264.7 cells. The activity of nuclear factor κB (NF-κB), a key regulator of inflammation, was markedly inhibited, contributing to the downregulation of inflammatory responses. Reduced activity of the MAPK signaling pathway was observed, indicating the potential role of Broadleaf Mahonia in modulating upstream inflammatory signaling cascades. Preventative treatment with Broadleaf Mahonia alleviated the symptoms of granulomatous lobular mastitis by reducing inflammation and promoting tissue homeostasis.	RAW264.7 cells	(125)
Schisandrin B (*Schisandra chinensis*)	Schisandrin B inhibited inflammation and ferroptosis and enhanced antioxidant response in *S. aureus*-induced mastitis Reduced the IL-1β, TNF-α, and MPO activities, enhanced GSH, GPX4 and Nrf2 levels. The Schisandrin B upregulated the SIRT1/p53/SLC7A11 signaling pathway, attenuating the activation of inflammation via suppressing the NF-κB activation	Mice	(126)
Gong Ying San	Anti-inflammatory, antibacterial, and antioxidant activities and reduced mastitis	Dairy Cows	(99)
*Prunella vulgaris* L.	Prevent LPS induced mastitis by inhibiting inflammatory markers including TNF-α, IL-6, and IL-1β, MAPK and NF-κB pathway-related proteins. In addition, upregulated the expressions of tight junction protein (ZO-1, occludin, and claudin-3) in mammary gland tissues.	Mice	(130)
*Angelica sinensis* Polysaccharide	Alleviates *S. aureus*-induced mastitis by enhancing gut microbiota in mice	Mice	(131)
Hordenine	Prevented lipopolysaccharide-induced mastitis by inhibiting inflammation through the TLR4-MAPK/NF-κB signaling pathway and reducing oxidative stress via the AMPK/Nrf2/HO-1 pathway, while also modulating the intestinal microbiota and preserving the blood–milk barrier by upregulating ZO-1, occludin, and claudin-3.	Mice	(132)
Iridoid glucosides	Suppressed the levels of TNF-α, IL-6, and IL-1β in *S. aureus*-stimulated mastitis in BMECs	BMECs	(133)
*Rhapontici Radix* extract (RRE)	RRE treatment reduced inflammatory cytokines (TNF-α, IL-6, and IL-1β) in LPS-induced mice mammary gland cells. The phosphorylation of MAPK and NF-κB pathways was reduced and upregulated the expression of TMEM59 and GPR161	MMECs	(134)
Pomegranate flower polysaccharid	Prevent mastitis by improving the intestinal flora in mice. Reduced inflammation by suppressing inflammatory cytokines	Mice	(135)
Quyu Xiaozhong	Quyu Xiaozhong treatment significantly reduced the pathological damage and inflammation in rats with *S. aureus*-induced mastitis. It decreased myeloperoxidase (MPO) activity and inflammatory factor levels TLR4, NF-κB-p65, and IκB-α. The inflammatory changes were inhibited via TLR4/NF-κB signaling pathway.	Rat	(136)
Saikosaponin A	Saikosaponin A significantly alleviated *S. aureus*-induced mastitis by reducing inflammation and preserving blood-milk barrier integrity. Inhibited NF-κB pathway activation and ferroptosis, characterized by reduced iron accumulation, mitochondrial changes, and enhanced antioxidant production. Upregulated key proteins involved in cellular protection, including SIRT1, Nrf2, HO-1, and GPX4.	Mice	(137)
Diosmetin	Diosmetin significantly reduced the pathological changes in the mammary gland caused by *S. aureus*. It decreased MPO activity, TNF-α and IL-1β release, and NF-κB activation. Diosmetin also inhibited *S. aureus*-induced malondialdehyde (MDA) and Fe2 + levels, while boosting ATP, glutathione (GSH) production, and GPX4 expression. Additionally, diosmetin upregulated SIRT1, Nrf2, and HO-1 expression	Mice	(138)
Allicin	Allicin, a natural extract from garlic, effectively reduced LPS-induced inflammation in MAC-T Treatment with 2.5 μM allicin significantly decreased the expression of pro-inflammatory cytokines IL-1β, IL-6, IL-8, and TNF-α, and inhibited the activation of the NLRP3 inflammasome. Allicin also suppressed the phosphorylation of IκB-α and NF-κB p65, indicating a blockade of NF-κB signaling. *In vivo*, allicin alleviated LPS-induced mastitis in mice, supporting its anti-inflammatory effects. These findings suggest that allicin mitigates mastitis by modulating the TLR4/NF-κB pathway, offering a potential alternative to antibiotics in dairy farming.	MAC-T	(139)
Chlorogenic acid	Chlorogenic acid demonstrated antimicrobial, antioxidant, and anti-inflammatory activities in combating *Escherichia coli* resistant induced mastitis. Killed *E.coli* by directly targeting bacterial cell well and membrane.	Cow	(140)
Chlorogenic acid extracted from *Taraxacum officinale*	Reduced the expression of pro-inflammatory genes and proteins such as TNF-α, IL-6, and IL-1β in lipoteichoic acid (LTA)-induced in BMECs. It also downregulated NO, TLR2, and NF-κB. Chlorogenic acid prevented inflammatory changes via suppressing TLR2/NF-κB pathway in BMECs.	BMECs	(141)
*Jiawei Yanghe* decoction	JYD significantly suppressed inflammatory changes and prevent mastitis in mice mammary gland via inhibiting TLR4/Myd88/NF-κB	Mice	(142)
Maslinic acid	Maslinic acid reduced pro-inflammatory factors (IL-6, IL-1β, TNF-α, iNOS, COX2), and protected the blood–milk barrier by maintaining tight-junction protein expression. Altered gut microbiota by promoting beneficial bacteria (*Enterobacteriaceae*) and inhibiting harmful bacteria (*Streptococcaceae*), Downregulated inflammation via suppressing NLRP3 inflammasome, AKT/NF-κB, and MAPK signaling pathways	Mice	(143)
Hexadecanamide	According to metabolomics analysis revealed that hexadecanamide was reduced in cows with SARA-associated mastitis. HEX alleviated *S. aureus*-induced mastitis in mice by suppressing inflammation and restoring the blood-milk barrier. *In vitro*, HEX inhibited NF-κB activation in mammary epithelial cells and activated PPARα, which upregulated SIRT1 to reduce inflammation.	MMECs	(144)
Wogonin	In LPS-treated mastitis models, wogonin reduced inflammatory cell infiltration, MPO activity, and pro-inflammatory cytokines (TNF-α, IL-1β), while inhibiting Akt/NF-κB pathway activation and enhancing Nrf2/HO-1 signaling. Wogonin also mitigated oxidative stress in MMECs by reducing ROS and MDA levels and increasing GSH and SOD levels.	MMECs	(145)
Anemoside B4 (*Pulsatilla chinensis*)	Prevented mastitis and inflammatory response by regulation lipid metabolism.	Cow	(146)
Oregano essential oil extracted from *Origanum vulgare*	Oregano essential oil suppressed key factors in dairy bovine mastitis, including TNF, TLR4, IL-1β, IL-6, IFNG, and MyD88, with major signaling pathways involving PI3K-Akt, MAPK, IL-17, and NF-κB.	Cow	(147)
Esculetin	Esculetin effectively mitigated inflammation in a murine model of mastitis induced by *S. uberis.* Reduced inflammatory cell infiltration and suppressed key inflammatory cytokines (IL-1β, IL-6, TNF-α). Mechanistically, inhibited P38 MAPK activation and NF-κB signaling, suggesting its therapeutic potential for mastitis management.	Mice	(148)
Retinoic acid	Alleviating low-grade endotoxemia-induced mammary injury and reducing proinflammatory cytokine production by suppressing the NF-κB/NLRP3 signaling pathway in MMECs Enhancing blood-milk barrier integrity through the upregulation of tight junction proteins, including ZO-1, Occludin, and Claudin-3	Mice	(149)
Evodiamine	Evodiamine alleviated mammary tissue injury, reduced pro-inflammatory cytokines, and suppressed inflammation-related pathways which suggesting its potential as a therapeutic agent for mastitis	Mice	(150)
Artemisinin	Improved cell viability and upregulated TLR4/NF-κB and MAPK/p38 signaling pathways in MAC-T cells. It also Reduced *E. coli*-induced inflammation by lowering TNF-α, IL-6, and IL-1β expression. In a mouse mastitis model, artemisinin alleviated mammary tissue damage, reduced inflammatory cell infiltration, and decreased inflammatory factor levels. These findings suggest that artemisinin may be an effective treatment for *E. coli*-induced mastitis.	Mice and MAC-T cells	(151)
Peiminine (alkaloid extracted from Fritillaria plants)	Protect against LPS-induced mastitis in mouse. Peiminine prevent inflammatory changes by inhibiting the phosphorylation of the protein kinase B (AKT)/ nuclear factor-κB (NF-κB), extracellular regulated protein kinase (ERK1/2), and p38 signaling pathways	Mice	(152)
Berberine hydrochloride	Berberine hydrochloride significantly reduced neutrophil infiltration and decreased the secretion and mRNA expression of TNF-α, IL-1β, and IL-6 in a dose-dependent manner. *Berberine hydrochloride* effectively suppressed LPS-induced activation of TLR4, NF-κB p65, and phosphorylation of I-κB. Berberine hydrochloride protects against LPS-induced mastitis by modulating the TLR4/NF-κB pathway.	Mice	(153)
Chinese Propolis (CP)	MAC-T cells treated with bacterial endotoxin (LPS), heat-inactivated *E.coli* and *S. aureus* showed significant decreases in cell viability, and inflammatory changes. Pretreatment with CP prevented the loss of cell viability. CP treatment resulted in decreased expressions of proinflammatory cytokine mRNAs, specifically IL-6 and TNF-α, compared to untreated mastitis-challenged cells. Enhanced expressions of antioxidant response by upregulating the key antioxidants genes via activation of Nrf2-ARE signaling pathway. CP demonstrated strong inhibitory effects against NF-κB activation, a key inflammatory transcription factor. The polyphenolic active components of CP, primarily caffeic acid phenethyl ester and quercetin, showed strong inhibitive effects against NF-κB activation.	MAC-T cells	(179)
Mangiferin	Mangiferin significantly alleviates LPS-induced histopathological changes in the mouse mastitis model Treatment with mangiferin decreases LPS-induced myeloperoxidase (MPO) activity Remarkably reduces the expression of pro-inflammatory cytokines TNF-α, IL-1β, and IL-6 Inhibits LPS-induced NF-ĸB signaling pathway activation Suppresses LPS-induced NLRP3 inflammasome activation The anti-inflammatory effects of mangiferin in LPS-induced mastitis are mediated through inhibition of both NF-ĸB and NLRP3 signaling pathways	Mice	(180)
Indirubin	Significantly attenuated the severity of inflammatory lesions, edema, inflammatory hyperemia, milk stasis, local tissue necrosis, and neutrophil infiltration in the LPS-induced mouse mastitis model Treatment significantly decreased myeloperoxidase activity in the mastitis model Downregulated the production of pro-inflammatory cytokines tumor necrosis factor-α, interleukin-1β, and IL-6 caused by LPS In vitro studies showed dose-dependent inhibition of LPS-stimulated expression of proinflammatory cytokines LPS-induced TLR4 expression was downregulated Treatment inhibited phosphorylation of LPS-induced NF-κB P65 protein and inhibitor of kappa B The MAPK signaling pathway was suppressed by inhibiting phosphorylation of extracellular signal-regulated kinase (ERK), P38, and c-jun NH2-terminal kinase (JNK) Anti-inflammatory effects were achieved through suppressing TLR4 and downstream NF-κB and MAPK pathway inflammatory signals	mice	(181)

HuMEC, Human mammary epithelial cells; BMECs, Bovine mammary epithelial cells.

While plant-derived bioactive compounds show promising antimicrobial and anti-inflammatory effects against bovine mastitis pathogens *in vitro* and in mouse models (128), their use in dairy cattle remains limited due to several inherent barriers in large animal research. Research on Traditional Chinese Medicine and plant-derived compounds for mastitis treatment is still in its early stages, with most trials still in development using mouse models, resulting in insufficient foundational data to support progress to large animal studies. Conducting controlled clinical trials in dairy cattle involves significant economic challenges, requiring much larger sample sizes, longer observation periods, and higher operational costs compared to mouse models, often surpassing available research budgets.

The regulatory framework governing veterinary pharmaceuticals in food-producing animals requires comprehensive safety evaluations, including pharmacokinetic studies, tissue residue analyses, and the establishment of withdrawal periods for milk and meat products, leading to lengthy approval processes that deter initial research investments. The physiological complexity of ruminant digestive systems adds further challenges, as plant-derived compounds undergo extensive ruminal metabolism that can alter bioavailability and therapeutic effectiveness compared to monogastric models. Additionally, dairy industry stakeholders usually prioritize rapid-acting, standardized antimicrobial treatments that work with existing automated milking protocols and quality systems, which creates market resistance to traditional plant-based therapies that need more complex preparation, administration, and monitoring. These economic, regulatory, physiological, developmental, and practical factors collectively explain why the translation of Traditional Chinese Medicine and plant-derived compounds from promising laboratory results to field use in dairy cattle is limited, despite their demonstrated anti-inflammatory, antimicrobial, and immunomodulatory properties in experimental studies (129).

## 6 Conclusion and future perspective

Based on available literature, we concluded that TCM and plant-derived bioactive compounds present a sustainable and effective alternative to conventional antibiotics for managing mastitis, addressing critical challenges such as antimicrobial resistance, drug residue in milk, and environmental impact. Plant-derived bioactive compounds and TCM have demonstrated efficacy in targeting key inflammatory and immune pathways (e.g., NF-κB, PI3K-AKT, MAPK) and improving milk quality without inducing remedies, which holds significant promise. Therefore, Plant- derived bioactive compounds and TCM require future efforts and concentration to elucidate its molecular mechanisms, standardize formulations, and conduct large-scale clinical trials to validate its efficacy and safety. Integrative approaches that combine plant- derived bioactive compounds and TCM with conventional therapies and advanced technologies, such as omics and artificial intelligence, can enhance therapeutic precision. Collaboration among researchers, policymakers, and farmers is essential to ensure scalability, farmer acceptance, and the establishment of harmonized regulatory frameworks, ultimately promoting plant-derived bioactive compounds and TCM as a mainstream, eco-friendly solution for mastitis control.
